# Familial colorectal cancer: search for novel predisposition genes

**DOI:** 10.1186/s40246-025-00901-y

**Published:** 2025-12-30

**Authors:** Asta Försti, Beiping Miao, Abhishek Kumar, Dagmara Dymerska Zaremba, Magdalena Marciniak, Jan Lubinski, Kari Hemminki

**Affiliations:** 1 Hopp Children’s Cancer Center (KiTZ), Heidelberg, Germany; 2 Division of Pediatric Neurooncology, German Cancer Research Center (DKFZ), German Cancer Consortium (DKTK), Im Neuenheimer Feld 580, 69120 Heidelberg, Germany; 3 Division of Immune Regulation in Cancer, German Cancer Research Center (DKFZ), Heidelberg, Germany; 4 State Key Laboratory of Oncogenes and Related Genes, Shanghai Cancer Institute, Renji Hospital, Shanghai Jiao Tong University School of Medicine, Shanghai, China; 5 Clinical Cooperation Unit Molecular Hematology/Oncology, German Cancer Research Center (DKFZ), Heidelberg, Germany; 6 Department of Genetics and Pathology, International Hereditary Cancer Center, Pomeranian Medical University in Szczecin, Szczecin, Poland; 7 Chair of Drug and Cosmetics Biotechnology, Warsaw University of Technology, Warsaw, Poland; 8 Biomedical Center, Faculty of Medicine, Charles University Pilsen, Alej Svobody 1665/76, 32300 Pilsen, Czech Republic; 9 Division of Cancer Epidemiology, German Cancer Research Center (DKFZ), Heidelberg, Germany

**Keywords:** Familial cancer, Multiple primaries, Colorectal cancer, Genetic predisposition

## Abstract

**Background:**

Family history of colorectal cancer (CRC) and multiple primary CRCs in a single person may indicate inherited CRC predisposition.

**Methods:**

In the present study, we performed whole exome/genome sequencing on germline DNA from at least two CRC cases in 19 families and from family members with a double primary CRC from seven additional families. We used a set of in silico predictions in combination with a STRING protein–protein interaction and pathway analysis to identify the most likely variants predisposing to CRC.

**Results:**

We identified Cell cycle/DNA repair and TGFβ signaling/Focal adhesion/Extracellular matrix organization pathways as highly significant protein–protein interaction networks. Variants in the *APCDD1*, *CYBA*, *PTK7* and *SRC* genes were identified in more than one family, and they were shown to dysregulate basic cellular functions, potentially leading to cancer development. Most variants were private to a family, and each family had more than one candidate variant, suggesting a synergistic or polygenic mode of inheritance. This hypothesis, as well as validation of the identified variants and pathways and their functional consequences, need confirmation by other family-based studies.

**Conclusions:**

Different types of family-based analyses together with in silico predictions are helpful to identify candidate genes and pathways for CRC predisposition.

**Supplementary Information:**

The online version contains supplementary material available at 10.1186/s40246-025-00901-y.

## Background

Family history of colorectal cancer (CRC) and multiple primary CRCs in a single person may indicate inherited CRC predisposition. Familial risk of CRC increases from 1.70 when one family member is diagnosed with CRC to 2.76 when two or more family members are diagnosed [1]. Familial CRC also increases the risk of double primary CRCs, up to 5 times compared to the sporadic disease [2]. The well-established high-risk CRC predisposition genes include the mismatch repair (MMR) genes *MSH2*, *MLH1*, *MSH6* and *PMS2* causing Lynch syndrome, and the Wnt-signaling gene *APC* causing familial adenomatous polyposis syndrome. In addition, variants in the base-excision repair gene *MUTYH* are causing recessively inherited adenomatous polyposis syndrome. Other well-characterized genes associated to dominantly inherited polyposis syndromes include TGFβ-pathway genes *SMAD4* and *BMPR1A*, in addition to the AMP-activated protein kinase *STK11* [3]. During the last 10 years, genome-wide sequencing has promoted studies attempting to identify novel high-to-moderate cancer predisposition genes [3]. These attempts have been successful in identifying genes predisposing to CRC polyposis syndromes, including polymerase proofreading genes *POLE* and *POLD1* [4], base excision repair gene *NTHL1* [5], MMR gene *MSH3* [6], TGFβ-pathway gene *GREM1* [7] and Wnt-pathway gene *RNF43* [8]. However, variants in these genes are rare, and they do not explain familial clustering of non-polyposis CRC.

In Sweden, about 15% of CRC patients have a first degree relative diagnosed with CRC [1], but according to a UK sequencing study of familial patients only about one quarter were explained by variants in the known high-penetrance genes [9]. Recently, genome-wide sequencing efforts, including our own, have attempted to fill in this gap and they have provided evidence for novel high-to-moderate penetrance CRC predisposition genes [3, 10–15]. Most of these genes need confirmation in additional families as well as functional validation of the identified variants. Here, we summarize and report our published and unpublished data on potential CRC predisposition genes after using an update of our Familial Cancer Variant Prioritization Pipeline (FCVPP) [16] in combination with a STRING protein–protein interaction and pathway analysis [17] on 19 CRC families, from which at least two cases were available for whole exome/genome sequencing (WES/WGS) and on seven additional CRC families presenting with double primary CRCs. We provide a list of all genes and variants prioritized by our pipeline to help scientists in the CRC predisposition community in further validation.

## Materials and methods

### Population

In the West-Pomeranian region of Poland, about 1.25 million (~ 70%) inhabitants participated in a population screening that was performed mainly in years 2000–2001. Cancer family history was collected from the participants and persons with positive CRC family history were invited to outpatient clinics of the Hereditary Cancer Center, Department of Genetics and Pathology, Pomeranian Medical University, Szczecin, Poland and their family histories were collected through face-to-face detailed interviews. An average review took 20–30 min. Similarly, persons with negative cancer family history were interviewed. Eligible individuals were asked to participate to the study and they signed an informed consent. Altogether, 1705 unrelated familial CRC cases and 1674 healthy elderly individuals without family history of cancer agreed to participate to the study and provided a blood sample for germline variant analysis. The study participants were screened for MMR gene mutations using multiplex ligation-dependent probe amplification (MLPA) and denaturing high-performance liquid chromatography (DHPLC), and more recently using a Polish HiRisk next generation sequencing (NGS) panel for mutations in the genes *APC, ATM, BRCA1, BRCA2, CDH1, CDKN2A, CHEK2, MLH1, MUTYH, MSH2, MSH6, NBN, PALB2, PTEN, PMS2, RAD51C, RAD51D, STK11, TP53*.

For the WES/WGS study, only families negative for the known cancer predisposition mutations based on the methodology used at the time of blood sample collection and initial screening were considered. From 19 families, at least two family members who were affected by CRC agreed to participate as described earlier [14]. Additionally, seven independent index cases with double primary CRCs were included in the study as described [15]. All these families had a prominent family history of CRC with the earliest age at diagnosis ranging from 23 to 64 years (Supplementary Fig. 1).

### Whole exome/genome sequencing (WES/WGS)

Germline DNA extracted from peripheral blood samples was used for WES/WGS. WES of families F1–F5 was performed using the Agilent SureSelect V5 with UTR target capture kit on the Illumina HiSeq 2000 platform and WGS of families F6–F15 and FA-FD was performed using the Illumina TrueSeq Nano DNA kit on the Illumina HiSeqX10 V2.5 platform at the DKFZ core facility as described [10, 14]. We converted the variant positions from the human reference genome GRCh37/hg19 to GRCh38/hg38 using the Assembly Converter of Ensembl release 114 -May 2025. The seven independent double primary CRCs were whole-exome sequenced by BGI Genomics using the Illumina Agilent V6-based sequencing (https://www.bgi.com/global) and the reads were aligned to the GRCh38/hg38 human reference genome as described [15].

### Variant annotation and filtering

Variants were annotated and filtered using an update of our in-house developed FCVPP [16]. We annotated the identified single nucleotide variants (SNVs) and small insertion/deletions (InDels) that were present in all affected family members of each family or in the double primary CRC cases using the Ensembl Variant Effect Prediction (VEP) tool [18]. We included only variants affecting the Ensembl canonical protein coding transcript. Minor allele frequency (MAF) filter of 0.1% was used with respect to gnomAD_exomes_AF, gnomAD_exomes_NFE_AF and gnomAD_exomes_POPMAX_AF data to remove common variants (gnomAD v.4.0.0; https://gnomad.broadinstitute.org). Rare SNVs and InDels ranking within the top 1% of potentially deleterious variants in the human genome were selected using the Combined Annotation Dependent Depletion (CADD v.1.7) tool; a scaled PHRED-like CADD score greater than 20 was applied [19].

### Variant prioritization: missense variants

We used several in silico tools to identify the most likely pathogenic missense variants. We screened the variants for their potential deleteriousness by using 11 different prediction tools: Sorting Intolerant from Tolerant (SIFT) [20], Polymorphism Phenotyping version 2 (PolyPhen-2) [21], Log ratio test (LRT) [22], MutationTaster [23], Mutation Assessor [24], Functional Analysis Through Hidden Markov Models (FATHMM) [25], MetaSVM [26], MetaLR [26], Protein Variation Effect Analyzer (PROVEAN) [27], AlphaMissense [28] and REVEL [29]. Variants predicted to be deleterious by > 50% of these tools were selected. To evaluate whether the variants are located in an evolutionary conserved position, we used three tools: Genomic Evolutionary Rate Profiling (GERP > 2.0) [30], PhastCons (> 0.3) [31] and Phylogenetic P-value (PhyloP ≥ 3.0) [32], with an inclusion cutoff of at least two positive predictions. To evaluate whether the affected genes are intolerant to variation, we report the Z-score, developed by the gnomAD consortium for missense variants [33]. Positive Z-scores indicate increased constraint, i.e. intolerance to variation.

### Loss-of-function variant analysis

Stop-gain, frameshift and splice-site variants affecting the canonical splice sites were considered when the CADD score criterion of > 20 was met. Variants affecting the last exon of the gene were excluded from further analyses, all other variants are reported. In order to discriminate pathogenic and neutral variants, we used MutPred2 (http://mutpred.mutdb.org) [34]. For each variant, it renders a score between zero and one; higher scores denote variants that are more likely to be pathogenic; a conservative threshold score of 0.50 at 5% false positive rate is recommended. MutPred2 also shows structural and functional mechanisms that are impacted in the affected region of the protein, accompanied by significant prior-corrected *P* values. Splice site variants were analyzed by using SpliceAI [35] and MMSplice [36] within the CADD v.1.7 tool. The SpliceAI Δ score ≥ 0.5 indicates a confidently predicted cryptic splice variant and Δ score ≥ 0.8 indicates a high-scoring predicted cryptic splice variant. The MMSplice score (absolute value) > 2 indicates a high confidence level prediction and > 1.5 medium level confidence. For LoF variants we report the LOEUF (loss-of-function observed/expected upper bound fraction) score from gnomAD which reflects the gene constraint; gnomAD recommends to use LOEUF < 0.6 as an indication of increased gene constraint, if a cut-off is needed, although many tumor suppressor genes, such as the MMR genes have a higher LOEUF.

### Network analysis with STRING

Protein–protein interactions within a biologically relevant pathway may give information about the importance of the identified genes in CRC development. We used the in silico tool STRING [17] to investigate the interactions between the proteins encoded by the prioritized genes and protein–protein interaction (PPI) enrichment within the created network. To create the network we used experiments, databases and co-expression as active interaction sources with a minimum required interaction score 0.400. In STRING, the PPI enrichment analysis is based on the input data compared to the random list of proteins. Such an enrichment indicates that the proteins are at least partially biologically connected, as a group. To calculate the false discovery rate (FRD), STRING uses the Benjamini–Hochberg procedure. We visualized the networks as clusters of interacting proteins in order to analyze functions that are specific to each cluster. For that purpose, we used Markov clustering (MCL) [37] as suggested by STRING and we report corresponding functional description of the clusters and the genes within the clusters. We also investigated pathway enrichment of the proteins within the two largest interacting networks based on Biological Processes within Gene Ontology (GO), Kyoto Encyclopedia of Genes and Genomes (KEGG) and Reactome. Further information of genes, proteins and their function were collected using GeneCards (https://www.genecards.org) and UniProt (https://www.uniprot.org). PubMed (https://pubmed.ncbi.nlm.nih.gov) was used to search for information about the relationship between the genes with cancer, especially in the context of CRC.

## Results

### Families and variant prioritization

**Fig. 1:**
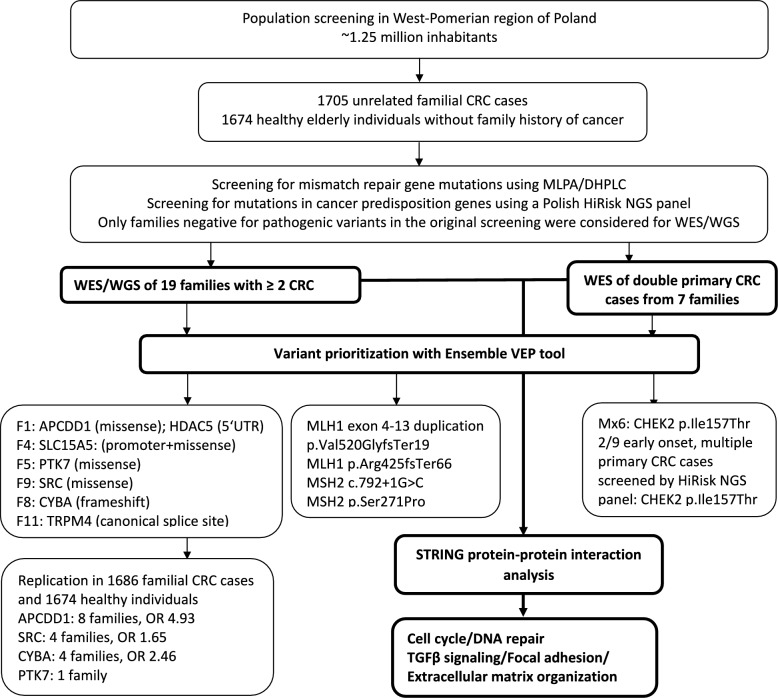
Workflow of the study. The integrated analysis of all variants prioritized using the Ensembl VEP analysis and the STRING protein–protein interaction analysis as the main focus of the study are highlighted in bold

The workflow of our study is shown in Fig. 1. We searched for rare, potentially pathogenic variants in 19 independent families, from which at least two CRC cases were sequenced using WES or WGS, and in seven independent cases with a double primary CRC and a family history of CRC using WES (Supplementary Fig. 1). The families were previously screened for pathogenic variants in known CRC predisposition genes using methodologies available at the time of recruitment of the CRC cases. When we screened the WES/WGS data for known CRC predisposition genes [38], we found three families with nonsense mutations in the MMR genes, including a large duplication of exons 4–13 causing an amino acid change p.Val520GlyfsTer19, a frameshift variant (p.Arg425SerfsTer66) and a canonical splice donor variant in intron 4, respectively [39]. We also found a missense MSH2 variant p.Ser271Pro in one additional family. In one double primary CRC case, Mx6, we found a moderate penetrance variant in CHEK2, p.Ile157Thr [15].

**Fig. 2:**
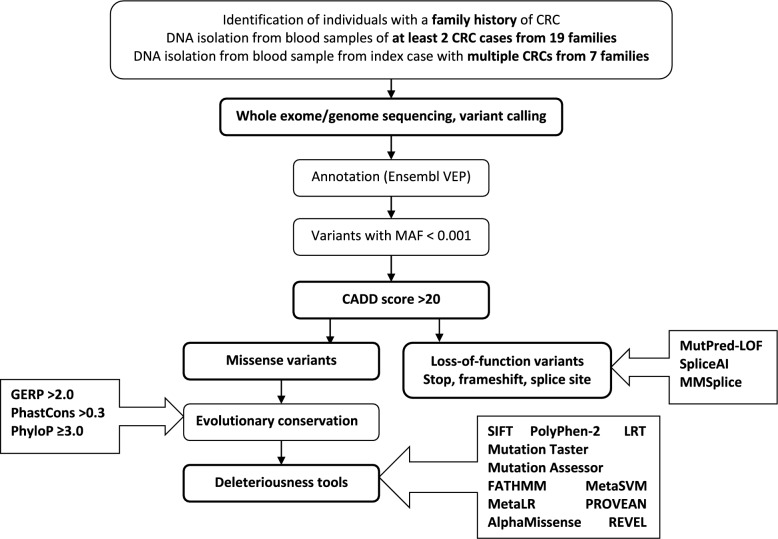
Pipeline for identification of the germline variants potentially predisposing to CRC

In our primary analysis for identification of novel CRC predisposition genes we excluded the families with the MMR gene mutations. We prioritized the variants that segregated with CRC in the 15 remining families or that were present in the double primary CRC cases using the pipeline shown in Fig. 2. We identified altogether 258 variants that passed the criteria of the pipeline, including 177 missense (89 in families/88 in double primary CRC cases), 31 stop-gain (17/14), 13 canonical splice site (3/10) and 37 frameshift (15/22) variants (Supplementary Tables 1 and 2). In the families F1-F15, there were between 1 (F3) and 22 (F12) variants per family; in the double primary CRC cases, the numbers were slightly higher, between 11 (Mx46) and 27 (Mx7).

### STRING protein–protein interaction and pathway analysis

**Fig. 3:**
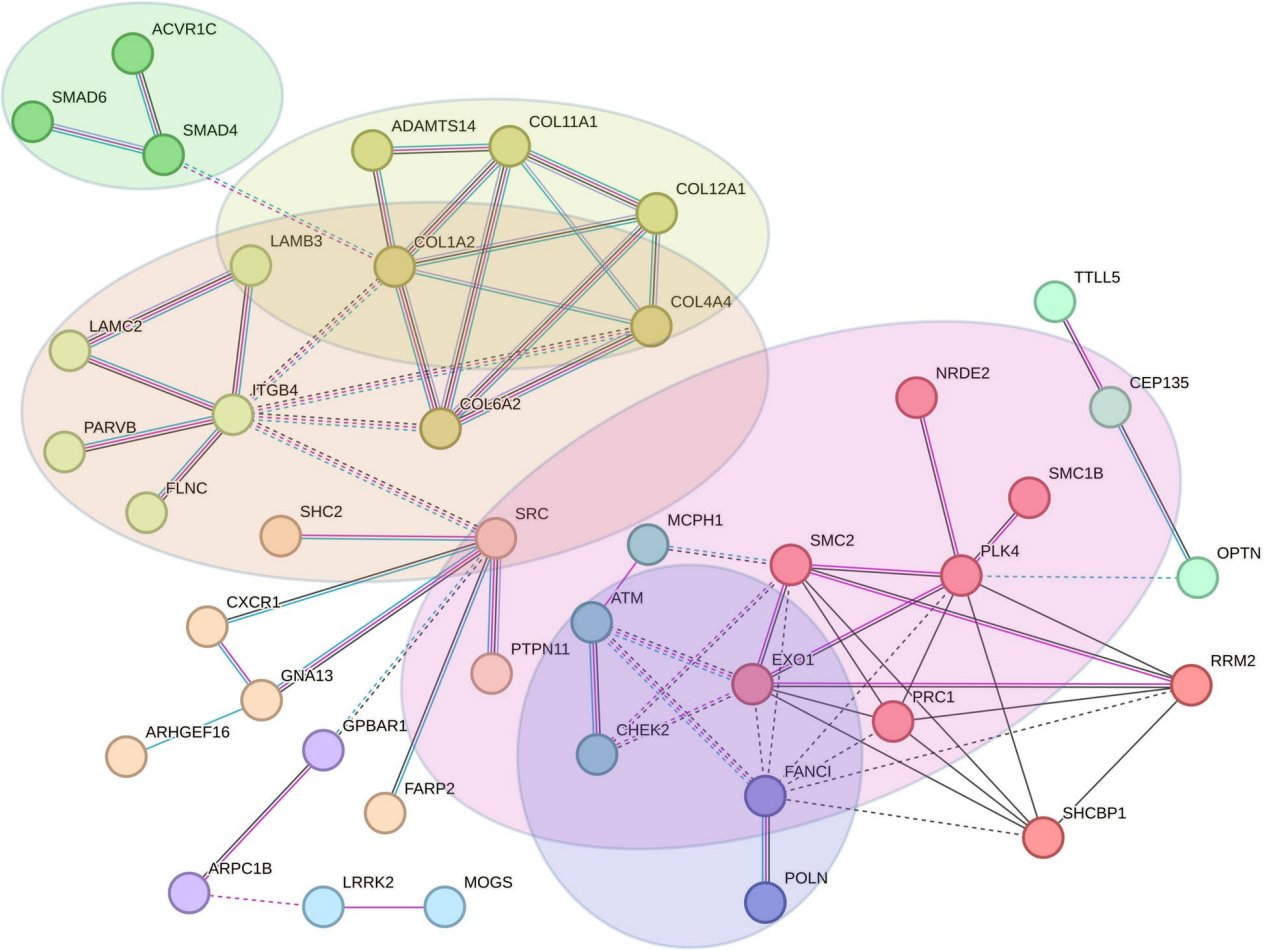
STRING protein–protein interaction networks of proteins encoded by the prioritized genes in 15 families and seven double primary CRC cases. Only the two largest networks are shown. Purple line between the nodes indicates experimental evidence of interaction between the proteins, light blue line indicates database evidence and black line co-expression evidence. Proteins related to KEGG pathway “TGFβ signaling” are shown in a green circle, “Focal adhesion” in an orange circle and GO Biological process “Extracellular matrix organization” in a yellow circle. Proteins related to GO Biological process “Cell cycle” are shown in a pink circle and Reactome pathway “DNA repair” in a blue circle

To further prioritize the most likely candidates for CRC predisposition genes, we combined the gene lists from the families and the double primary CRC cases, and used the corresponding protein list as an input for the STRING protein–protein interaction (PPI) analysis. Altogether 91 proteins interacted with at least one other protein (PPI enrichment *P* value 6.33 × 10^−7^) (Supplementary Fig. 2). MCL clustering analysis identified 32 clusters (Supplementary Table 3). From these, six clusters were connected to each other in a large interaction network related to KEGG pathways “TGFβ signaling”, “Focal adhesion” and GO Biological process “Extracellular matrix organization” (Fig. 3). More specifically, the MCL algorithm-based clusters were related to “Signaling by Activin” (ACVR1C, SMAD4, SMAD6), “Collagen biosynthesis and modifying enzymes” (COL1A2, COL4A4, COL6A2, ADAMTS14, COL12A1, COL11A1), “Cell junction organization” (ITGB4, PARVB, FLNC, LAMC2, LAMB3), “Netrin mediated repulsion signals” (SRC, FARP2, SHC2, CXCR1, GNA13, PTPN11, ARHGEF16) and two undetermined two-protein clusters (ARPC1B, GPBAR1 and LRRK2, MOGS) (Table 1). Another larger network was related to GO Biological process “Cell cycle” and Reactome pathway “DNA repair” (Fig. 3) and included the MCL algorithm-based clusters “SMC proteins Flexible Hinge Domain” (PLK4, RRM2, SHCBP1, NRDE2, SMC1B, SMC2, EXO1, PRC1), “DNA damage induced protein phosphorylation” (ATM, MCPH1, CHEK2), “Fanconi Anemia Pathway” (FANCI, POLN) and one unclassified cluster of three proteins (CEP135, OPTN, TTLL5) (Table 1). Both the proteins identified in the families and in the double primary CRC cases contributed to these large networks, as shown in Supplementary Fig. 2.

**Table 1:** Description of genes and variants in the main STRING clusters

Family_ID	Gene	Gene name	Chrom_Pos_Ref_Alt	Ensemble transcript; HGVSc	HGVSp	CADD_phred	Deleteriousness score n/11*	MutPred2_LOF score	MutPred2 mechanisms	Protein function
Family 1	FLNC	Filamin C	7_128840655_G_A	ENST00000325888.13:c.1657G > A	ENSP00000327145.8:p.Gly553Ser	25.5	9			Actin cytoskeleton organization; sarcomere organization
	PLK4	Polo Like Kinase 4	4_127896849_GT_G	ENST00000270861.10:c.2754del	ENSP00000270861.5:p.Val919CysfsTer42	33		0.43	NA	Centriole replication during cell cycle
Family 5	GNA13	G Protein Subunit Alpha 13	17_65053567_T_C	ENST00000439174.7:c.445A > G	ENSP00000400717.2:p.Ile149Val	20.8	7			Transmembrane signaling
Family 6	LAMB3	Laminin Subunit Beta 3	1_209630647_G_A	ENST00000356082.9:c.911C > T	ENSP00000348384.3:p.Pro304Leu	27.6	7.5			Cell adhesion; cell migration
	CXCR1	C-X-C Motif Chemokine Receptor 1	2_218164374_G_A	ENST00000295683.3:c.838C > T	ENSP00000295683.2:p.Arg280Cys	25.4	6.5			IL8 signaling; immune response
Family 7	ATM	ATM Serine/Threonine Kinase	11_108301732_G_T	ENST00000675843.1:c.5262G > T	ENSP00000501606.1:p.Lys1754Asn	23	6			DNA damage response (DSB repair)
Family 8	COL6A2	Collagen Type VI Alpha 2 Chain	21_46114063_G_A	ENST00000300527.9:c.791G > A	ENSP00000300527.4:p.Arg264His	32	8			Cell adhesion
Family 9	SRC	SRC Proto-Oncogene, Non-Receptor Tyrosine Kinase	20_37394253_G_A	ENST00000373578.7:c.529G > A	ENSP00000362680.2:p.Val177Met	31	9			Cell adhesion; cell cycle; immune response
Family 12	COL11A1	Collagen Type XI Alpha 1 Chain	1_102995999_C_T	ENST00000370096.9:c.2285G > A	ENSP00000359114.3:p.Arg762Gln	28.9	9			Extracellular matrix protein
	EXO1	Exonuclease 1	1_241889544_G_T	ENST00000366548.8:c.2485G > T	ENSP00000355506.3:p.Glu829Ter	43		0.50	NA	DNA mismatch repair; immune response
Family 14	CEP135	Centrosomal Protein 135	4_56019469_A_C	ENST00000257287.5:c.3129A > C	ENSP00000257287.3:p.Lys1043Asn	23.4	6			Centriole biogenesis
	PARVB	Parvin Beta	22_44131621_G_A	ENST00000338758.12:c.511G > A	ENSP00000342492.6:p.Val171Met	26.9	7.5			Cell adhesion; cell migration
	POLN	DNA Polymerase Nu	4_2229168_G_A	ENST00000511885.6:c.64C > T	ENSP00000435506.1:p.Gln22Ter	37		NA	NA	DNA repair; homologous recombination
Family 15	ARHGEF16	Rho Guanine Nucleotide Exchange Factor 16	1_3477968_CGGCCAACGTGCTACCTTTTCCTGTT_C	ENST00000378378.9:c.1568_1592del	ENSP00000367629.4:p.Arg523ProfsTer7	35		0.31	NA	Unknown; protein–protein and protein–lipid interactions
Mx6	LAMC2	Laminin Subunit Gamma 2	1_183227656_C_T	ENST00000264144.5:c.1427C > T	ENSP00000264144.4:p.Thr476Met	23.7	6.5			Cell adhesion
	CHEK2	Checkpoint Kinase 2	22_28725099_T_C	ENST00000404276.6:c.470 T > C	ENSP00000385747.1:p.Ile157Thr	23.5	6.5			Cell cycle, DNA repair
	SMAD4	SMAD Family Member 4	18_51058218_CTGGT_C	ENST00000342988.8:c.762_765del	ENSP00000341551.3:p.Gly255SerfsTer80	29.5		0.69	NA	Tumor suppressor; TGFbeta signaling
	SHC2	SHC Adaptor Protein 2	19_460954_CG_C	ENST00000264554.11:c.42del	ENSP00000264554.4:p.Ala15ArgfsTer164	23		0.41	Methylation (*p* = 0.037); Amidation (*p* = 0.044)	Signal transduction
Mx7	COL12A1	Collagen Type XII Alpha 1 Chain	6_75102661_G_T	ENST00000322507.13:c.8351C > A	ENSP00000325146.8:p.Pro2784His	27	8			Cell adhesion; ECM organization
	SMC1B	Structural Maintenance Of Chromosomes 1B	22_45372273_C_T	ENST00000357450.9:c.2078G > A	ENSP00000350036.4:p.Arg693His	24.6	7			Cell cycle; meiosis
	RECQL	RecQ like helicase	12_21486541_G_A	ENST00000444129.7:c.439C > T	ENSP00000416739.2:p.Gln147Ter	38		0.61	Magnesium binding (*p* = 0.036); Amidation (*p* = 0.040)	DNA repair
	SHCBP1	SHC binding and spindle associated 1	16_46604439_G_A	ENST00000303383.8:c.712C > T	ENSP00000306473.3:p.Arg238Ter	36	0.4	NA	–	Cell proliferation, growth and differentiation
Mx8	COL4A4	Collagen Type IV Alpha 4 Chain	2_227007353_C_T	ENST00000396625.5:c.5045G > A	ENSP00000379866.3:p.Arg1682Gln	27.1	8			ECM organization
	NRDE2	NRDE-2, Necessary For RNA Interference, Domain Containing	14_90292787_C_G	ENST00000354366.8:c.1752G > C	ENSP00000346335.3:p.Glu584Asp	23.8	6.5			Cell cycle; DNA damage response; mRNA processing
	PRC1	Protein Regulator Of Cytokinesis 1	15_90979162_A_C	ENST00000394249.8:c.1103 T > G	ENSP00000377793.3:p.Phe368Cys	32	7.5			Cell cycle
	SMAD6	SMAD Family Member 6	15_66703558_TGGGAGCTCCCTGCTGGACGTGGCGGAGCC_T	ENST00000288840.10:c.306_334del	ENSP00000288840.5:p.Ser102ArgfsTer9	28.3		0.53	NA	BMP and TGF-beta/activin-signaling
Mx29	RRM2	Ribonucleotide Reductase Regulatory Subunit M2	2_10128903_C_T	ENST00000304567.10:c.854C > T	ENSP00000302955.4:p.Ser285Leu	22.9	7			Cell cycle; deoxyribonucleotide synthesis
Mx34	ACVR1C	Activin A Receptor Type 1C	2_157556276_G_C	ENST00000243349.13:c.361C > G	ENSP00000243349.7:p.Pro121Ala	21.5	6			TGFbeta signaling
	ARPC1B	Actin Related Protein 2/3 Complex Subunit 1B	7_99389957_G_T	ENST00000646101.2:c.445G > T	ENSP00000496599.1:p.Asp149Tyr	32	7			Actin polymerization; transcription regulation; DNA repair
	SMC2	Structural Maintenance Of Chromosomes 2	9_104118330_A_G	ENST00000374793.8:c.1951A > G	ENSP00000363925.3:p.Thr651Ala	26.1	10.5			Cell cycle; mitosis
	ADAMTS14	ADAM Metallopeptidase With Thrombospondin Type 1 Motif 14	10_70752124_C_T	ENST00000373207.2:c.2626C > T	ENSP00000362303.1:p.Arg876Cys	29.7	6.5			Collagen degradation
	LRRK2	Leucine Rich Repeat Kinase 2	12_40340400_G_A	ENST00000298910.12:c.6055G > A	ENSP00000298910.7:p.Gly2019Ser	28.2	10.5			Multiple processes, e.g. autophagy, differentiation; Wnt signaling
	PTPN11	Protein Tyrosine Phosphatase Non-Receptor Type 11	12_112477722_A_G	ENST00000351677.7:c.925A > G	ENSP00000340944.3:p.Ile309Val	23	6			Multiple cell functions, e.g. cell growth, differentiation, mitosis, oncogenic transformation
	OPTN	Optineurin	10_13112464_T_TAG	ENST00000378747.8:c.381_382insAG	ENSP00000368021.3:p.Asp128ArgfsTer22	22.3		0.30	Coiled coil (*p* = 0.022); Helix (*p* = 0.045); Carboxylation (*p* = 0.046)	Autophagy; innate immunity
	FARP2	FERM, ARH/RhoGEF And Pleckstrin Domain Protein 2	2_241468263_CCT_C	ENST00000264042.8:c.2021_2022del	ENSP00000264042.3:p.Leu674GlnfsTer31	26.6		0.39	Allosteric site (*p* = 0.037)	RAC signal transduction; cell adhesion
Mx36	MOGS	Mannosyl-Oligosaccharide Glucosidase	2_74461994_C_G	ENST00000448666.7:c.1795G > C	ENSP00000410992.3:p.Asp599His	26.2	8			N-linked oligosaccharide processing
	COL1A2	Collagen Type I Alpha 2 Chain	7_94427840_C_T	ENST00000297268.11:c.3481C > T	ENSP00000297268.6:p.Arg1161Cys	29.5	9			ECM organization
	ITGB4	Integrin Subunit Beta 4	17_75756515_C_T	ENST00000200181.8:c.4795C > T	ENSP00000200181.3:p.Arg1599Cys	31	6.5			Cell adhesion; cancer
	FANCI	FA complementation group I	15_89295009_C_T	ENST00000310775.12:c.2551C > T	ENSP00000310842.8:p.Gln851Ter	43		0.48	NA	DNA repair
	MCPH1	Microcephalin 1	8_6442069_T_TC	ENST00000344683.10:c.586dup	ENSP00000342924.5:p.Gln196ProfsTer8	25.5		0.32	Iron binding (*p* = 7.1352e − 05); Catalytic site (*p* = 0.0006); Disulfide linkage (*p* = 0.00156); Proteolytic cleavage (*p* = 0.005); Signal helix (*p* = 0.0055)	Chromosome condensation and DNA damage induced cellular responses
	TTLL5	Tubulin tyrosine ligase Like 5	14_75683658_T_C	ENST00000298832; HGVSc:c.371 + 2 T > C**		33				Transcription
Mx43	GPBAR1	G Protein-Coupled Bile Acid Receptor 1	2_218262944_CT_C	ENST00000519574.2:c.221del	ENSP00000430202.1:p.Leu74ArgfsTer42	25.1		0.30	NA	Bile acid receptor

^*^The tools used to evaluated the deleteriousness of the variants include SIFT, PolyPhen-2, LRT, Mutation Taster, Mutation Assessor, FATHMM, MetaSVM, MetaLR, Provean, Alphamissense and REVEL

^**^SpliceAI-don-loss 0.99; MMSp_donor -1.363

Most of the variants in the two main networks were missense variants (Supplementary Tables 1 and 2). However, both *SMAD4* (Mx6) and *SMAD6* (Mx8) variants affecting the TGFβ signaling pathway caused a frameshift, followed by premature termination of the translation. Within the Cell cycle/DNA repair pathway clusters, three stop-gain variants were identified: *EXO1* (F12), *FANCI* (Mx36) and *POLN* (F14). Furthermore, two frameshift variants leading to a premature stop codon were found: *MCPH1* (MX36) and *PLK4* (F1). In eight families, two or more variants within these pathways were prioritized, including F1 with the *FLNC* and *PLK4* variants, F12 with the *EXO1* and *COL11A1* variants, F14 with the *POLN*, *CEP135* and *PARVB* variants, Mx6 with the *CHEK2*, *SMAD4*, *LAMC2* and *SHC2* variants, Mx7 with 2 missense variants in *SMC1B* and *COL12A1*, Mx8 with the *SMAD6*, *PRC1*, *NRDE* and *COL4A4* variants, Mx34 with four missense variants in *ACVR1C*, *ADAMTS 14*, *PTPN11* and *SMC2*, and Mx36 with *FANCI*, *MCPH1*, *ITGB4* and *COL1A2* variants.

### Other potential predisposition genes

We found also other interesting genes that were related to the pathways identified by the STRING analysis, but not connected to the two main STRING networks. *ANKRD53* (stop-gain variant, CADD 39), *CDK18* (splice donor-loss variant, CADD 34), *KDM8* (CADD 29.2), *ZW10* (CADD 26.5), *TTC28* (CADD 33) and *TUBG2* (CADD 25.6) were among the genes related to cell cycle, and ENDOV (stop-gain variant, CADD 35), POLL (CADD 29.1) and RECQL (stop-gain variant, CADD 38) were related to DNA repair (Supplementary Tables 1 and 2). LTBP3 (CADD 25.3) and TGFBI (CADD 23.5) were related to TGFβ pathway and CDH4 (CADD 25.3), LGALS3BP (stop-gain variant, CADD 34) and PTK7 (CADD 22.7) to cell adhesion (Supplementary Tables 1 and 2).

Previously, we had done a detailed analysis, including basic functional tests, of six individual families and prioritized the Wnt-pathway gene *APCDD1* and the histone deacetylase *HDAC5* (5’UTR variant) in F1 [11], the innate immunity-related gene *SLC15A5* (a missense and a promoter variant) in F4 [13], the Wnt-pathway and cell adhesion gene *PTK7* in F5 [10], the proto-oncogene *SRC* in F9 [12], as well as *CYBA* (frameshift variant) in F8 and *TRPM4* (splice acceptor-loss variant) in F11 related to mucus biology and inflammation [14]. From the encoded proteins, only SRC was present in the main STRING networks (Fig. 3; Table 1). In addition to the *SRC* variant, which was found in altogether five families, the variant in *APCDD1* was found in nine families, the one in *CYBA* in five families and the one in *PTK7* in two families (Supplementary Table 4).

In our secondary analysis we included the genes prioritized by our pipeline in the four families with MMR gene mutation (Supplementary Table 5) to the above STRING analysis. The main contribution of the corresponding proteins to the PPI network was the modification of the Cell cycle/DNA repair cluster, in which MLH1 and MSH2 interacted with each other and with ATM, EXO1 and FANCI (Supplementary Fig. 3, Supplementary Table 6). MSH2 interacted additionally with CHEK2 and SMC2. MSH2 also added RECQL with a stop-gain variant from the double primary CRC case Mx7 to the DNA repair cluster, and connected the Cell cycle/DNA repair cluster to the TGFβ signaling/Focal adhesion/Extracellular matrix (ECM) organization cluster.

## Discussion

Only a small proportion of familial CRC is explained by established high-penetrance cancer predisposition genes. World-wide efforts using genome-wide sequencing approaches in CRC families have produced some success in identifying novel predisposition genes for CRC polyposis syndromes, and suggested candidate genes for familial CRC in general [3]. However, most of the candidates have been identified only in single families. In the present study, we combined the data from our WES/WGS studies on 26 CRC families, and identified Cell cycle/DNA repair and TGFβ signaling/Focal adhesion/ECM organization pathways as commonly affected across the families. The variants in the *APCDD1*, *CYBA*, and *SRC* genes were identified in more than one family with odds ratios ranging from 1.6 to 4.9; *PTK7* variant was found in another family with four CRC cases [10–12, 14]. Functional studies gave clues for their functional significance.

Among the families we sequenced, we identified three with an MMR gene mutation leading to a truncated protein; in one additional family the MMR gene mutation was a missense amino acid change with unknown significance. These mutations might have been missed due to the methodology, DHPLC, used in the early 2000 [39], highlighting the importance of re-screening historically negative cases with up-to-data technologies. Among the families, from which the double primary CRC case was sequenced, we found a moderate-penetrance variant *CHEK2* p.Ile157Thr; this family had also been screened using DHPLC [15]. Interestingly, previous screening using the HiRisk NGS panel had identified two other double primary CRC cases with the same variant, thus suggesting its involvement in CRC predisposition in this type of families [15].

In the Cell cycle/DNA repair pathway, we identified several genes which were affected by variants leading to protein truncation, including *EXO1*, *FANCI*, *POLN* and *RECQL* with a stop-gain variant and *MCPH1* and *PLK4* with a frameshift variant. Defects in DNA repair genes are common both in the hereditary and sporadic CRC, highlighted by the well-established role of germline mutations in the MMR genes in Lynch syndrome. Interestingly, our STRING PPI analysis showed both experimental, database and co-expression-based interactions between the MMR genes and genes in our Cell cycle/DNA repair cluster. From our candidate genes, *ATM*, *EXO1* and *RECQL* were among the genes expressed in the intestine with variants having a CADD score > 20 and MAF < 0.1% in a Swedish study on 55 CRC families [40]. One study has also reported a rare, homozygous missense variant in *ATM* in an early-onset CRC case, however, acknowledging the problems evaluating the pathogenicity of the large number of rare missense variants identified in *ATM* [41]. In addition to *RECQL*, other RecQ like helicases, including *WRN* and *BLM,* have been suggested as familial CRC predisposition genes [42, 43]. Also, enrichment of variants in Fanconi anemia DNA repair genes (*FANCC*, *FANCD1*, *FANCE*, *FANCJ)* in six out of 40 Spanish families has been reported [44] and a biallelic inactivation of *FANCM* in an advanced CRC case [45]. A study on 48 familial CRC cases from Australia identified a pathogenic stop-gain variant in *FANCI*, but did not prioritize the gene as it was not linked to cancer in cancer-associated databases COSMIC, OncoKB and TSGene [46]. That study also reported a stop-gain variant with unknown significance in a cancer-associated *FANCD2* gene. These studies give further confidence for the involvement of additional DNA repair genes in CRC predisposition.

In the TGFβ signaling/Focal adhesion /ECM organization pathway, frameshift variants were identified in the *SMAD4* and *SMAD*6 genes, as well as in the *SHC2* gene. From these, *SMAD4* is known to predispose to Juvenile polyposis syndrome [3], but it is also commonly mutated in sporadic CRC [47]. Similar to the SMAD proteins, SHC adaptor proteins are signal transducers mediating signals from growth factors, cytokines and integrins [48]. They have been linked to various diseases, including cancer, however, SHC2 seems to function mainly in the peripheral nervous system [48]. We identified also several missense variants in genes coding both major collagenous and non-collagenous constituents of basement membrane of the ECM, including collagens and laminins, and a missense variant in an integrin-coding gene, *ITGB4*, involved in the cell-ECM interactions. An abnormal TGFβ-ECM axis has been recognized as an important component in cancer development and progression, promoting cell proliferation, differentiation, survival and migration [49]. Our results highlight a role of germline variation in remodeling the ECM through the TGFβ-focal adhesion-ECM axis.

Some limitations should be considered when interpreting our results. For seven out of the 22 families without MMR mutations, the prioritization of the variants was based only on in silico tools with partly overlapping prediction algorithms. This strategy may have led to identification of false positives. However, for the other 15 families, segregation of the variants with CRC supported their potential pathogenicity, with functional characterization of the prioritized variants from five families. Also, the variants in *APCCD1*, *CYBA*, *PTK7* and *SRC* were found in other independent familial CRC cases. However, we acknowledge that although some of the families were large, we had only 2–4 samples from CRC patients from each family available for sequencing, and for the seven double primary CRC families only the index case was available. Additionally, we excluded the few healthy family members who agreed to participate to the study as they usually were from a younger generation, and thus uninformative for the segregation analysis. In the study, we performed both WES and WGS, and used tree different library preparation kits and sequencing platforms, which may have introduced some batch effects. However, as we focused on only coding germline variants, for which each allele should be present at about 50% frequency, the problem should be smaller than in the identification of somatic mutations.

## Conclusions

Our study identified the Cell cycle/DNA repair and TGFβ signaling/Focal adhesion/ECM organization as the main pathways affected by germline variants in 26 Polish families. Variants in the *APCDD1*, *CYBA*, *PTK7* and *SRC* genes were identified in more than one family, and they were shown to dysregulate basic cellular functions, potentially leading to cancer development. Most variants were private to a family, and each family had more than one candidate variant, thus suggesting a synergistic or polygenic mode of inheritance. This hypothesis, as well as validation of the identified variants, and pathways and their functional consequences, need confirmation by other family-based studies.

## Supplementary Information

Supplementary Material 1.

Supplementary Material 2.

Supplementary Material 3.

Supplementary Material 4.

Supplementary Material 5.

Supplementary Material 6.

Supplementary Material 7.

## Data Availability

The whole exome and whole genome sequencing data generated in this study are available in the European Genome-Phenome Archive (EGA) under accession numbers EGAS50000000606 and EGAS00001005118.
