# Supplementary material for: Familial colorectal cancer: search for novel predisposition genes
Source: Hum Genomics. 2025 Dec 30;20:22. doi: 10.1186/s40246-025-00901-y (PMC12849417; doi:10.1186/s40246-025-00901-y)
Supplement: Supplementary file 1 — Supplementary Material 1. [file 40246_2025_901_MOESM1_ESM.pdf]

SUPPLEMENTARY DATA

FAMILIAL COLORECTAL CANCER: SEARCH FOR NOVEL PREDISPOSITION GENES

Asta Försti[1,2,*], Beiping Miao[3,4], Abhishek Kumar[5], Dagmara Dymerska Zaremba[6,7], Magdalena Marciniak[6], Jan Lubinski[6] and Kari Hemminki[8,9,*]

Supplementary Material 1 is a pdf file including Supplementary figures 1-3 and their description and description of Supplementary Tables 1-6.

Supplementary Figure 1. Pedigrees of the 15 families (F1-15), from which at least two CRC case subjects were sequenced, the seven families (Mx6- Mx43), from which only the double CRC case was sequenced, and the four families, in which a mismatch repair gene mutation was identified (Family A: MLH1 exon 4-13 duplication; Family B: MLH1 p.Arg425SerfsTer66; Family C: MSH2 canonical splice site variant in intron 4; Family D: MSH2 p.Ser271Pro). The sequenced cases are shown by *. CRC cases in the families are shown in black symbols, all other cancer cases in light grey symbols, family members with polyps are also indicated. Col - Colorectal cancer; End - Endometrial cancer; Kid – Kidney cancer; Lar - Laryngeal cancer; Liv – Liver cancer; Lu - Lung cancer; Pan – Pancreatic cancer; Pr – Prostate cancer; Sto - Stomach cancer; Abd – Abdominal disease; CSU - Cancer site unknown; Fgt – Female genital tract; UC – Ulcerative colitis; d - died

Supplementary Figure 2. Protein-protein interaction networks of proteins encoded by the prioritized genes in 15 families and seven double primary CRC cases. Only connected nodes are shown. Each cluster identified by STRING with the MCL algorithm is shown in its own color as described in Supplementary Table 3. Purple line between the nodes indicates experimental evidence of interaction between the proteins, light blue line indicates database evidence and black line co-expression evidence. The clusters are organized to show proteins identified in the 15 families in light blue boxes and the proteins identified in the seven double primary CRC cases in light red boxes.

Supplementary Figure 3. Protein-protein interaction networks of proteins encoded by the prioritized genes in 15 CRC families combined with the prioritized genes in the double primary CRC patients and families with the MMR gene mutations. Only connected nodes are shown. Each cluster identified by STRING with the MCL algorithm is shown in its own color as described in Supplementary Table 6. Purple line between the nodes indicates experimental evidence of interaction between the proteins, light blue line indicates database evidence and black line co-expression evidence.

Supplementary Material 2-7 include Supplementary Tables 1-6 as Excel files.

Supplementary Table 1. Available as an Excel file. Overview of the variants identified in the 15 families (F1-F15), from which at least two CRC case subjects were sequenced. Genes within the two large main clusters are implicated in bold; those in the minor clusters are implicated in Italics. (A) Missense variants, (B) Stop-gain variants leading to truncated proteins, (C) Canonical splice site variants, (D) Frameshift variants leading to truncated proteins.

Supplementary Table 2. Available as an Excel file. Overview of the variants identified in the seven families (Mx6-Mx43), with the double primary CRC case sequenced. Genes within the two large main clusters are implicated in bold; those in the minor clusters are implicated in Italics. (A) Missense variants,

(B) Stop-gain variants leading to truncated proteins, (C) Canonical splice site variants, (D) Frameshift variants leading to truncated proteins.

Supplementary Table 3. Available as an Excel file. STRING analysis based on the MCL algorithm of the prioritized genes in our study of 15 CRC families combined with the prioritized genes in the double primary CRC patients. (A) Cluster description, (B) Cluster genes.

Supplementary Table 4. Available as an Excel file. Overview of the variants reported in our previous studies, including APCCD1, HDAC5, SRC, PTK7, CYBA, TRPM4 and SLC15A4.

Supplementary table 5. Available as an Excel file. Overview of variants prioritized by the pipeline in the families with MMR gene mutations.

Supplementary Table 6. Available as an Excel file. STRING analysis based on the MCL algorithm of the prioritized genes in our study of 15 CRC families combined with the prioritized genes in the double primary CRC patients and families with the MMR gene mutations. (A) Cluster description, (B) Cluster genes.

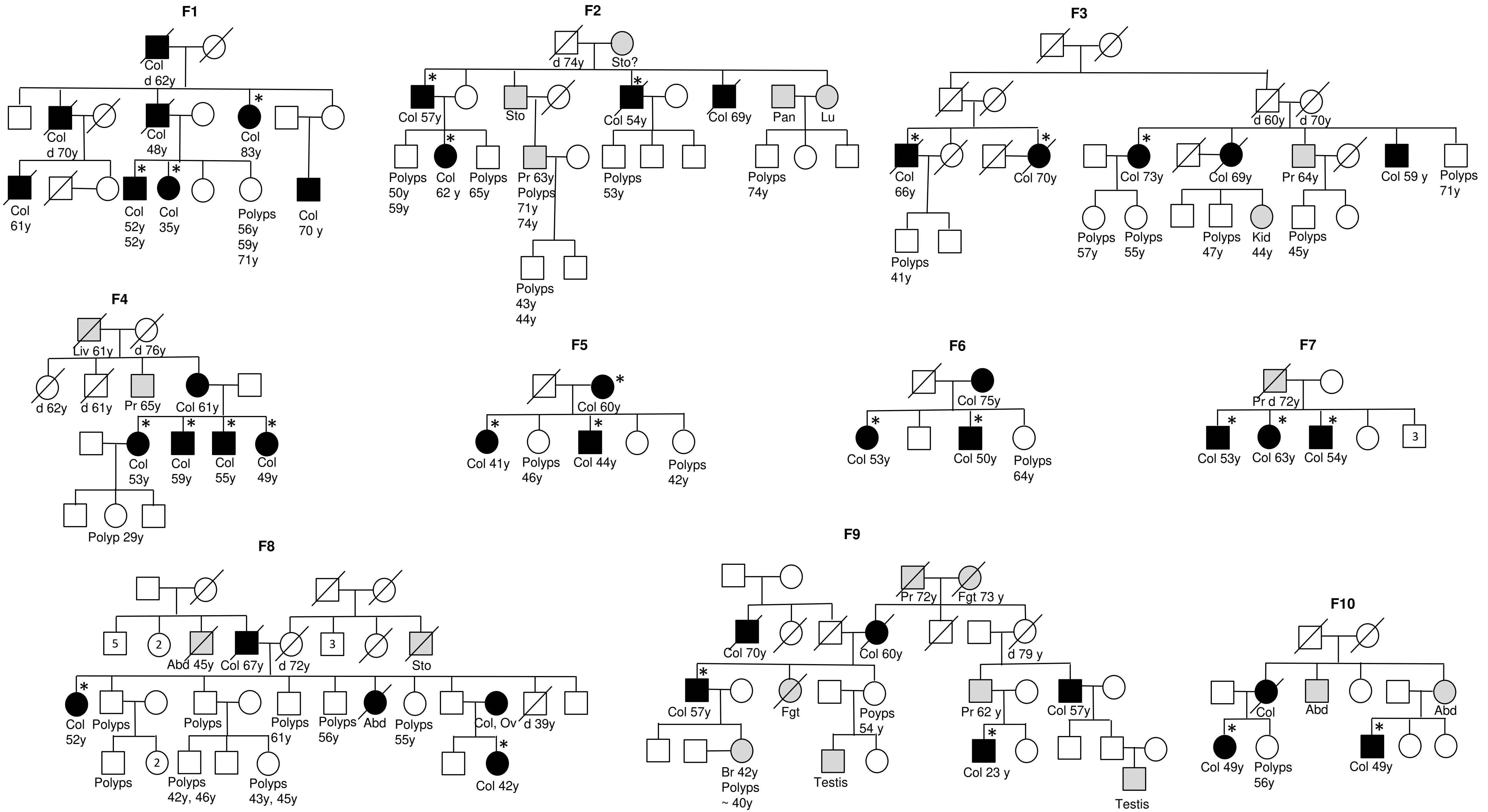

F1
Col
d 62y
Col
d 70y
Col
48y
*
Col
83y
Col
61y
*
Col
52y
52y
*
Col
35y
Polyps
56y
59y
71y
Col
70 y
F2
d 74y
Sto?
*
Col 57y
Sto
*
Col 54y
Col 69y
Pan
Lu
Polyps
50y
59y
*
Col
62 y
Polyps
65y
Pr 63y
Polyps
71y
74y
Polyps
53y
Polyps
74y
Polyps
43y
44y
F3
d 60y
d 70y
*
Col
66y
*
Col 70y
*
Col 73y
Col 69y
Pr 64y
Col 59 y
Polyps
71y
Polyps
57y
Polyps
55y
Polyps
47y
Kid
44y
Polyps
45y
Polyps
41y
F4
Liv 61y
d 76y
d 62y
d 61y
Pr 65y
Col 61y
*
Col
53y
*
Col
59y
*
Col
55y
*
Col
49y
Polyp 29y
F5
*
Col 60y
*
Col 41y
Polyps
46y
*
Col 44y
Polyps
42y
F6
Col 75y
*
Col 53y
*
Col 50y
Polyps
64y
F7
Pr d 72y
*
Col 53y
*
Col 63y
*
Col 54y
3
F8
5
2
Abd 45y
Col 67y
d 72y
3
Sto
*
Col
52y
Polyps
Polyps
Polyps
61y
Polyps
56y
Abd
Polyps
55y
Col, Ov
d 39y
Polyps
2
Polyps
42y, 46y
Polyps
43y, 45y
*
Col 42y
F9
Pr 72y
Fgt 73 y
Col 70y
Col 60y
d 79 y
*
Col 57y
Fgt
Poyps
54 y
Pr 62 y
Col 57y
Br 42y
Polyps
~ 40y
Testis
*
Col 23 y
Testis
F10
Col
Abd
Abd
*
Col 49y
Polyps
56y
*
Col 49y

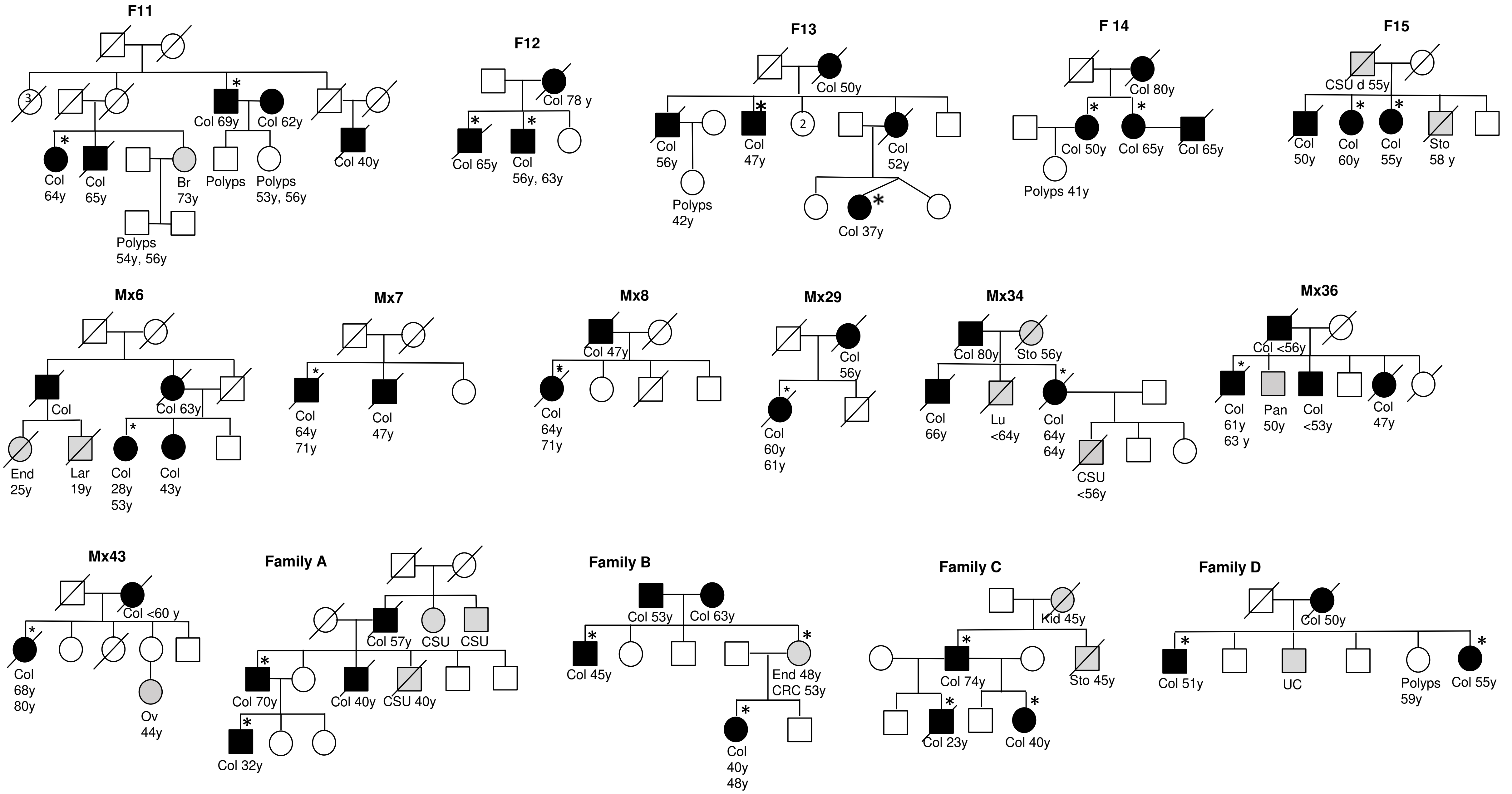

F11
3
*
Col 69y
Col 62y
Col 40y
Col 64y
Col 65y
Br 73y
Polyps
Polyps 53y, 56y
Polyps 54y, 56y
F12
Col 78 y
Col 65y
Col 56y, 63y
F13
Col 50y
2
Col 56y
Col 47y
Col 52y
Polyps 42y
Col 37y
F 14
Col 80y
Col 50y
Col 65y
Col 65y
Polyps 41y
F15
CSU d 55y
Col 50y
Col 60y
Col 55y
Sto 58 y
Mx6
Col
Col 63y
End 25y
Lar 19y
Col 28y 53y
Col 43y
Mx7
Col 64y 71y
Col 47y
Mx8
Col 47y
Col 64y 71y
Mx29
Col 56y
Col 60y 61y
Mx34
Col 80y
Sto 56y
Col 66y
Lu <64y
Col 64y 64y
CSU <56y
Mx36
Col <56y
Col 61y 63 y
Pan 50y
Col <53y
Col 47y
Mx43
Col <60 y
Col 68y 80y
Ov 44y
Family A
Col 57y
CSU
CSU
Col 70y
Col 40y
CSU 40y
Col 32y
Family B
Col 53y
Col 63y
Col 45y
End 48y
CRC 53y
Col 40y 48y
Family C
Kid 45y
Col 74y
Sto 45y
Col 23y
Col 40y
Family D
Col 50y
Col 51y
UC
Polyps 59y
Col 55y

Supplementary Figure 1. Pedigrees of the 15 families (F1-15), from which at least two CRC case subjects were sequenced, the seven families (Mx6-Mx43), from which only the double CRC case was sequenced, and the four families, in which a mismatch repair gene mutation was identified (Family A: MLH1 exon 4-13 duplication; Family B: MLH1 p.Arg425SerfsTer66; Family C: MSH2 canonical splice site variant in intron 4; Family D: MSH2 p.Ser271Pro). The sequenced cases are shown by *. CRC cases in the families are shown in black symbols, all other cancer cases in light grey symbols, family members with polyps are also indicated. Col - Colorectal cancer; End - Endometrial cancer; Kid – Kidney cancer; Lar - Laryngeal cancer; Liv – Liver cancer; Lu - Lung cancer; Pan – Pancreatic cancer; Pr – Prostate cancer; Sto - Stomach cancer; Abd – Abdominal disease; CSU - Cancer site unknown; Fgt – Female genital tract; UC – Ulcerative colitis; d - died

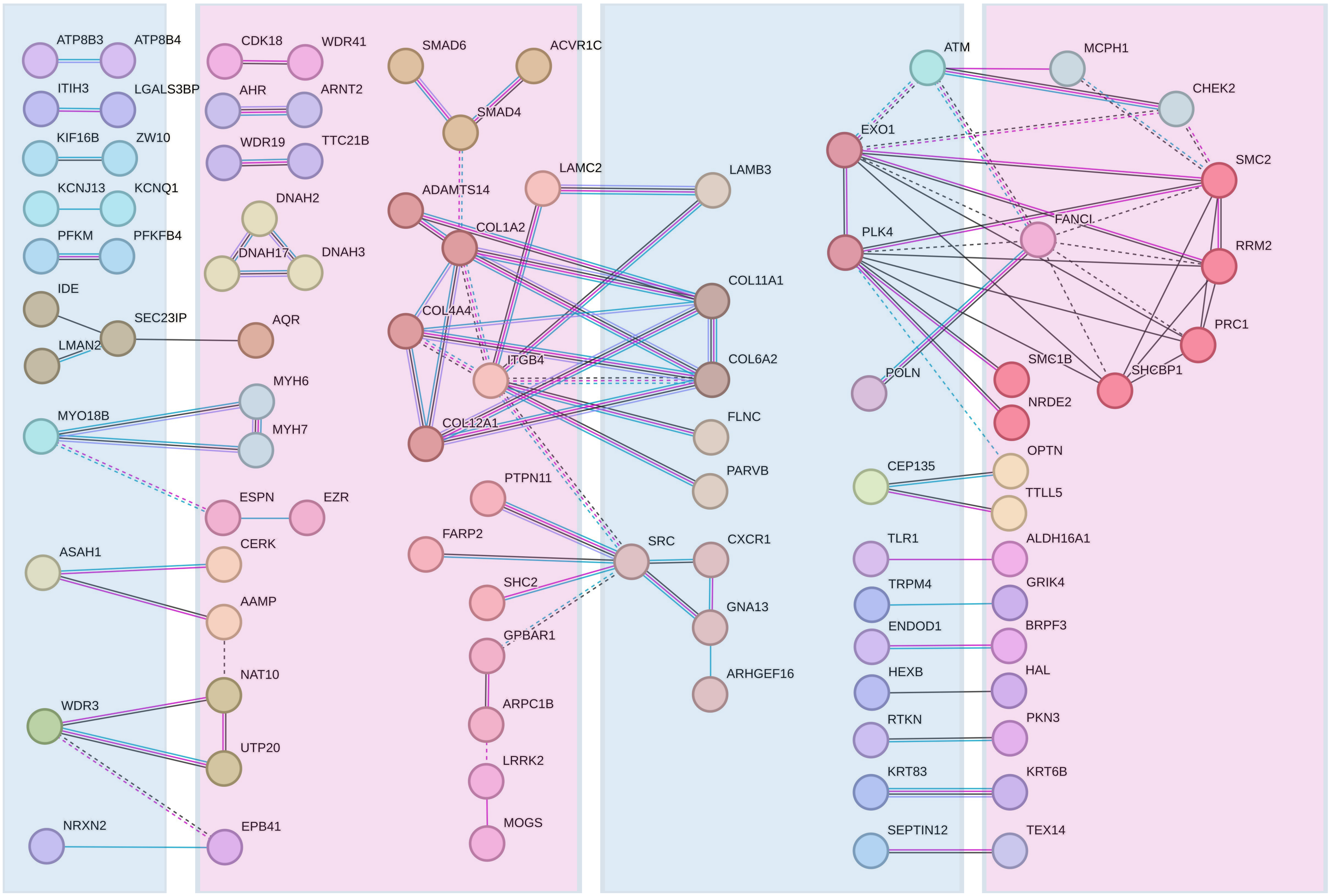

ATP8B3
ATP8B4
ITIH3
LGALS3BP
KIF16B
ZW10
KCNJ13
KCNQ1
PFKM
PFKFB4
IDE
SEC23IP
LMAN2
AQR
MYO18B
MYH6
MYH7
ESPN
EZR
ASAH1
CERK
AAMP
NAT10
WDR3
UTP20
NRXN2
EPB41
CDK18
WDR41
AHR
ARNT2
WDR19
TTC21B
DNAH2
DNAH17
DNAH3
SMAD6
ACVR1C
SMAD4
ADAMTS14
LAMC2
COL1A2
COL4A4
ITGB4
COL12A1
PTPN11
FARP2
SHC2
GPBAR1
ARPC1B
LRRK2
MOGS
LAMB3
COL11A1
COL6A2
FLNC
PARVB
SRC
CXCR1
GNA13
ARHGEF16
ATM
MCPH1
CHEK2
EXO1
SMC2
FANCI
PLK4
RRM2
PRC1
SMC1B
SHCBP1
POLN
NRDE2
OPTN
CEP135
TTLL5
TLR1
ALDH16A1
TRPM4
GRIK4
ENDOD1
BRPF3
HEXB
HAL
RTKN
PKN3
KRT83
KRT6B
SEPTIN12
TEX14

Supplementary Figure 2. Protein-protein interaction networks of proteins encoded by the prioritized genes in 15 families and seven double primary CRC cases. Only connected nodes are shown. Each cluster identified by STRING with the MCL algorithm is shown in its own color as described in Supplementary Table 3. Purple line between the nodes indicates experimental evidence of interaction between the proteins, light blue line indicates database evidence and black line co-expression evidence. The clusters are organized to show proteins identified in the 15 families in light blue boxes and the proteins identified in the seven double primary CRC cases in light red boxes.

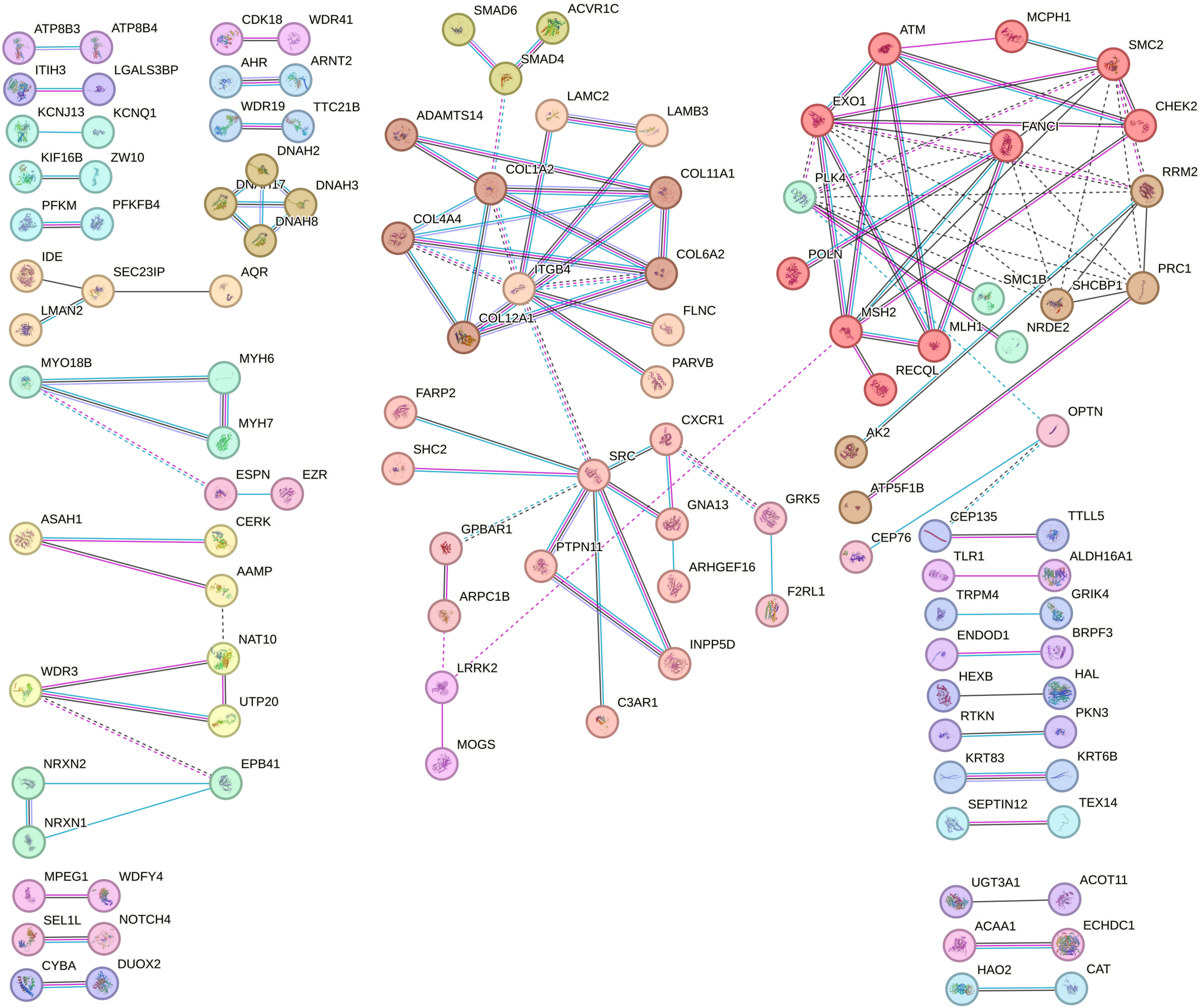

ATP8B3
ATP8B4
ITIH3
LGALS3BP
KCNJ13
KCNQ1
KIF16B
ZW10
PFKM
PFKFB4
IDE
SEC23IP
AQR
LMAN2
MYO18B
MYH6
MYH7
ESPN
EZR
ASAH1
CERK
AAMP
NAT10
WDR3
UTP20
NRXN2
EPB41
NRXN1
MPEG1
WDFY4
SEL1L
NOTCH4
CYBA
DUOX2
CDK18
WDR41
AHR
ARNT2
WDR19
TTC21B
DNAH2
DNAH3
DNAH8
SMAD6
ACVR1C
SMAD4
LAMC2
LAMB3
ADAMTS14
COL1A2
COL11A1
COL4A4
COL6A2
ITGB4
COL12A1
FLNC
PARVB
FARP2
CXCR1
SHC2
SRC
GRK5
GNA13
GPBAR1
PTPN11
ARHGEF16
ARPC1B
F2RL1
INPP5D
LRRK2
C3AR1
MOGS
ATM
MCPH1
SMC2
EXO1
CHEK2
FANCI
PLK4
RRM2
POLN
SMC1B
SHCBP1
PRC1
MSH2
MLH1
NRDE2
RECQL
AK2
OPTN
ATP5F1B
CEP135
TTLL5
CEP76
TLR1
ALDH16A1
TRPM4
GRIK4
ENDOD1
BRPF3
HEXB
HAL
RTKN
PKN3
KRT83
KRT6B
SEPTIN12
TEX14
UGT3A1
ACOT11
ACAA1
ECHDC1
HAO2
CAT

Supplementary Figure 3. Protein-protein interaction networks of proteins encoded by the prioritized genes in 15 CRC families combined with the prioritized genes in the double primary CRC patients and families with the MMR gene mutations. Only connected nodes are shown. Each cluster identified by STRING with the MCL algorithm is shown in its own color as described in Supplementary Table 6. Purple line between the nodes indicates experimental evidence of interaction between the proteins, light blue line indicates database evidence and black line co-expression evidence.
